# A Review of Advancing Two-Dimensional Material Membranes for Ultrafast and Highly Selective Liquid Separation

**DOI:** 10.3390/nano12122103

**Published:** 2022-06-18

**Authors:** Hongli Zhang, Yiling Zheng, Shuwen Yu, Weixing Chen, Jie Yang

**Affiliations:** 1 School of Materials Science and Chemical Engineering, Xi’an Technological University, Xi’an 710021, China; zhengyiling0522@163.com (Y.Z.); chenwx@xatu.edu.cn (W.C.); 2 Key Laboratory of Spin Electron and Nanomaterials of Anhui Higher Education Institutes, School of Chemistry and Chemical Engineering, Suzhou University, Suzhou 234000, China; swyu917@163.com; 3 School of Materials Science and Engineering, Xi’an Polytechnic University, Xi’an 710048, China

**Keywords:** two-dimensional nanomaterials, separation membrane, graphene oxide, MXene, metal organic frameworks, covalent organic frameworks

## Abstract

Membrane-based nanotechnology possesses high separation efficiency, low economic and energy consumption, continuous operation modes and environmental benefits, and has been utilized in various separation fields. Two-dimensional nanomaterials (2DNMs) with unique atomic thickness have rapidly emerged as ideal building blocks to develop high-performance separation membranes. By rationally tailoring and precisely controlling the nanochannels and/or nanoporous apertures of 2DNMs, 2DNM-based membranes are capable of exhibiting unprecedentedly high permeation and selectivity properties. In this review, the latest breakthroughs in using 2DNM-based membranes as nanosheets and laminar membranes are summarized, including their fabrication, structure design, transport behavior, separation mechanisms, and applications in liquid separations. Examples of advanced 2D material (graphene family, 2D TMDs, MXenes, metal–organic frameworks, and covalent organic framework nanosheets) membrane designs with remarkably perm-selective properties are highlighted. Additionally, the development of strategies used to functionalize membranes with 2DNMs are discussed. Finally, current technical challenges and emerging research directions of advancing 2DNM membranes for liquid separation are shared.

## 1. Introduction

Owing to overpopulation and rapid industrialization, water scarcity has evolved to be one of the most important issues that influences global economic development [1,2]. Wastewater and seawater have long been considered as potential sources from which to produce fresh water [3]. Compared with conventional water treatment techniques, such as adsorption, electrochemical treatment, distillation, and thermal desalination, the membrane-based separation process is considered as one of most promising and extensively adopted methods for combining the superiorities of high separation efficiency, low economic and energy consumption, small footprint, continuous operation mode, and environmental friendliness [4,5]. However, membranes are subject to a trade-off between selectivity (to what extent the desired molecules are separated from the rest) and permeability (how fast molecules pass through a membrane material) [6]. Advances in developing membranes with both fast flux and high selectivity have been hindered by the inherent limitations of the traditional membrane materials used and relatively empirical, unchangeable membrane processing strategies [7]. An ideal membrane should be as thin as possible to maximize permeation, have a narrow pore size distribution to enable efficient separation, and also be mechanically, chemically, and thermally robust for a long service life.

In recent years, various two-dimensional nanomaterials (2DNMs), including the graphene family, layered transition metal dichalcogenides (TMDCs), metal–organic frameworks (MOFs), covalent organic frameworks (COFs) and MXene (2D transition metal carbide and/or nitride) nanosheets have been increasingly explored as superb building blocks to develop high-performance molecular separation membranes [8,9,10,11]. Unlike common membranes based on polymeric and inorganic materials, 2DNMs with unique mono-atomic thickness enable the formation of an ultrathin membrane-separating layer, which minimizes transport resistance and maximizes permeation flux, offering ultimate separation capabilities [12,13]. Table 1 compares the differences in properties of 2D material membranes and their advantages and disadvantages in water treatment. Inorganic membranes using zeolites or MOFs exhibit relatively excellent separation capabilities due to their precisely defined pore apertures and chemical functionalities. Unfortunately, owing to high engineering cost, uncontrolled defect formation, and poor reproducibility, the economically large-scale production of zeolites or MOF membranes is still a major technical challenge in practical separation applications [14,15]. Recently, the carbon-based 2DNM of graphene (and its derivatives) has attracted considerable interest in membrane-based liquid separation. This 2DNM shows intrinsically uniformly sized apertures or drilled nanopores which enable 2DM-based membranes to achieve highly specific separation [16]. Moreover, 2D nanomaterials can be controllably assembled into laminates with a predefined interlayer distance, which acts as a molecular sieving channel. By rationally tailoring and precisely controlling the in- and out-of-plane nanopore structures and/or chemical functionalities, these membranes constructed from 2D materials can generate perfect molecular sieving membranes for unprecedented molecular separation properties [17,18].

Some reviews have wonderfully described the development of 2D material-based membranes in various separation fields, especially graphene and GO membranes. However, the latest progress, future trends, existing challenges and opening opportunities with regard to membranes derived from the large family of 2D materials have not been reviewed. Therefore, in this review, we aim to present a timely overview of 2DNM-based membranes for rapid and efficient liquid separation. We firstly explain the separation and transport mechanisms of molecules and/or ions through 2DM-based membranes and summarize the development of strategies for processing the 2DNMs into prospective separation membranes. On this basis, a variety of new and advanced 2DNMs used for membrane designs are discussed at length. We focus on the current state-of-the-art fabrication methods, nanostructures designs, membrane performances, and applications in various liquid separations. Finally, current technical challenges and emerging research directions for advancing 2DNM membranes for liquid separation will be additionally discussed. These 2DNM-based membranes exhibit ultra-fast and highly selective transport properties, opening a new avenue to develop next-generation high-performance separation membranes.

## 2. Separation Mechanisms of 2DNM Membranes

**Figure 1:**
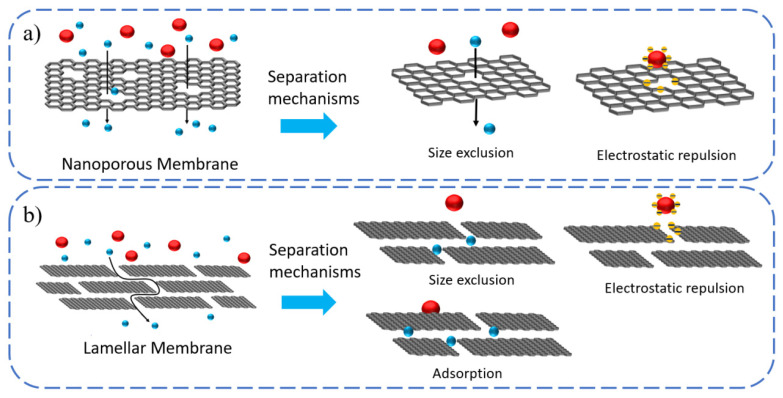
Schematic representation of the two types of 2DNM-based membranes. (**a**) Nanoporous membranes consist of a single layer of 2D material flakes with nanopores of precisely defined pore size. Selectivity is achieved by size exclusion and electrostatic repulsion between the charged species and the pores. (**b**) Membranes composed of stacked 2D material flakes. In stacked 2DNM membranes, the size of the pores is determined by the interlayer spacing between the flakes. In addition to size exclusion and electrostatic interaction, selectivity in-stacked 2DNM-based membranes also result from ion adsorption via electrostatic binding.

Based on their atomic structure, 2D materials are either porous or nonporous. Thereby, they can be processed into two forms of separation membranes: nanosheet and laminar membranes (Figure 1). Typically, the former consists of single or few layers of 2D materials with intrinsic precisely defined nanopores (e.g., COFs, MOFs) or accurate perforation (graphene) for selective permeation. The latter is formed by the controllable assembly of 2DNM nanosheets (such as graphene oxide, GO) into layered stacks with interlayer channels providing molecule/ion transport. Nanopores can be created on monolayer 2D nanosheets by ion beam bombardment, plasma etching, oxidation etching, electron beam irradiation, etc. [19]. By contrast, the stacking of 2D flakes could form uniform interlayer spacing that is comparable in size to ions or molecules. To achieve higher permeability, the stacking distance of these laminates can also be effectively increased by inserting the nanomaterials into the interlayer of the 2DM or crosslinking with macromolecules [20,21].

The separation and interception of 2DNM-based membranes are mainly achieved based on two main mechanisms, including size exclusion and the electrostatic interaction/Donnan effect [22]. According to the pore-size-induced size-exclusion mechanism, 2DNM-based membranes with evenly sized nanopores are able to reject molecules with sizes larger than the 2DM in-plane pores, while small-sized separating species can easily pass through. Pore-chemistry-induced electrostatic repulsion has great effects on membrane separation performance. When the surface of the 2DNM-based membrane is negatively charged, it can hinder the passage of negatively charged molecules or ions, and simultaneously facilitate the migration of positively charged molecules or ions through electrostatic interactions [22]. In addition to pore and interlayer channel separation mechanisms, functional groups decorated on the surfaces of 2D materials are also critically important in molecular and ion separations because they can selectively interact with each other to facilitate or hinder permeation. Hence, selectivity in stacked 2DNM-based membranes also results from the adsorption of ionic species to the 2D material flakes via electrostatic binding, cation π and metal coordination, etc. By precisely tuning the interlayer spacing and nanopores structures and rationally optimizing the surface chemical functionalities, the use of 2DMs for membrane designs can effectively improve permeance and selectivity.

## 3. 2D Nanosheet Membranes (Porous Membranes)

Nanosheets of 2DNMs with evenly sized nanopores can be used as membranes for ultimate separation. Membrane pores are created either by intrinsic porous structures, such as in MOFs and COFs, or by drilled pores such as in graphene-like materials. The permeation flux and selectivity of nanosheet membranes are determined by the membrane thickness and pore structures. Hence, by varying the size and geometry of the monomers, the sizes of pores within these materials could be rationally tuned, which would allow the fabrication of porous membranes for specific separation. In this section, we will focus on porous sheet membranes with regard to the issues mentioned above.

### 3.1. MOF

Metal–organic frameworks (MOFs) are a family of porous crystalline materials composed of inorganic metal ions or metal clusters connected by organic ligands via coordination bonds [23,24]. Thanks to the advantages of high surface area, regular and highly tunable pore structure, and functional surface groups, MOF-based membranes are regarded as a superior candidate for liquid separation [25]. It still remains a great challenge to achieve ultrathin 2D metal–organic frameworks nanosheets. Two strategies are mainly employed for synthesizing 2D MOF nanosheets: top-down [26] and bottom-up methods [27]. In the top-down strategy, Liu’s group [26] prepared 2D nanosheets by weakening the interlayer van der Waals forces of the 3D layered MOF through intercalated H^+^ ions and the electrostatic interaction of negatively charged congo red (CR) dye. This exfoliation method yielded ultrathin monolayer (3.4 nm) 2D Zn-MOF nanosheets with extra-large lateral dimensions (~6 mm). A new bottom-up strategy [27] was adopted to synthesize zeolitic imidazolate framework-67 (ZIF-67) nanosheets. ZIF-67 nanosheets were prepared by 2-methylimidazole and Co^2+^ in the mixed solvent of DMF and H_2_O at room temperature.

**Figure 2:**
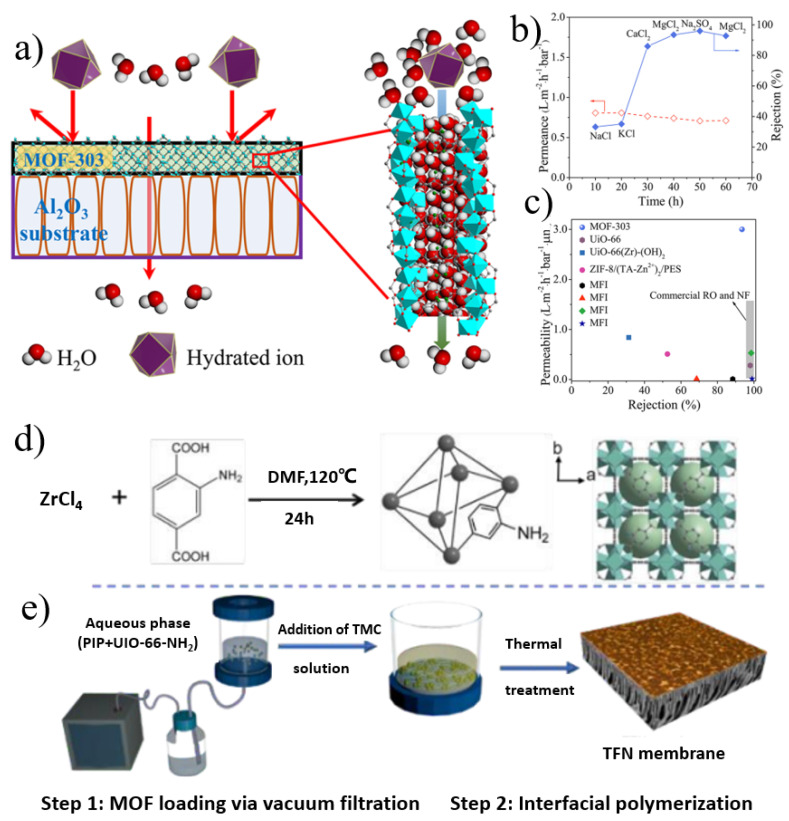
(**a**) Schematic representation of water desalination with a metal−organic framework MOF-303 membrane. (**b**) MOF-303 membrane separation performance for water desalination. (**c**) Desalination (Mg^2+^ retention) performances of MOF-303, zeolite, commercial polymeric reverse osmosis (RO), nanofiltration (NF), and other MOF membranes. (**d**) Synthesis of UiO-66-NH_2_ nanocrystals. (**e**) Fabrication of thin-film MOF-positioned PA membranes assisted by the filtration of the aqueous phase prior to interfacial polymerization. Reprinted with permission from [28,29]. Copyright (2021) American Chemical Society. Copyright (2019) Royal Society of Chemistry.

The tunable and controllable structure and excellent separation performance of MOF-containing membranes make them promising scavengers for water pollution control, such as the removal of heavy metal ions, the removal of organic dye pollution, and the desalination of brackish water and seawater. For the preparation of MOF nanosheet-based films, the deposition of MOF nanosheets on porous substrates and the incorporation of MOF nanosheets into polymers are the two main approaches so far. Cong and co-workers [28] fabricated continuous polycrystalline MOF-303 films on porous α-Al_2_O_3_ discs via an in situ hydrothermal synthesis method (Figure 2a–c). The surface of the MOF-303 membrane is well-intergrown crystals without obvious cracks. Based on the size-sieving mechanism and electrostatic repulsion effect, the membrane achieved an extraordinary rejection for divalent ions (93.5% for MgSO_4_ and 96.0% for Na_2_SO_4_) with unprecedented permeability (3.0 L·m^−2^·h^−1^·bar^−1^·μm). The low material cost and good stability indicate that MOF-303 is a promising new-generation membrane material for desalination.

By combining multifunctional organics with nanofiltration membranes by different methods, nanofiltration membranes with high selectivity and permeability have been prepared which are expected to play an important role in sewage treatment. In another study [29], MOF crystals were synthesized as functional nanofillers to fabricate polyamide (PA) composite membrane (Figure 2d,e). Amino-functionalized UiO-66 MOFs were synthesized via H_2_bdc-NH_2_ and ZrCl_4_ under solvothermal conditions. The thin-film nanocomposite membrane was first assisted by vacuum filtration of the aqueous phase (UiO-66-NH_2_, piperazine), and then interfacially polymerized with trimesoyl chloride (TMC). The terminal amino groups of Zr-MOFs formed a covalent bond with TMC, which tightly bound the MOF crystals to PA. The surface of the composite membrane was a rough fish mesh structure; the introduction of MOF not only increased the membrane surface area but also created more transmission channels. The TFN membrane exhibited a favorable Na_2_SO_4_ rejection of 97.5% and superior water permeability (30.8 L·m^−2^·h^−1^·bar^−1^). This filtration-assisted controllable loading of hydrophilic MOFs provides a useful approach to fabricate TFN membranes with outstanding separation performance. Wen et al. [30] developed a superselective polyamide (PA) membrane by enhancing the interfacial polymerization of amphiphilic MOF nanoflakes. The created ultrathin nanofilms had an intrinsic thickness of about 5 nm and a degree of crosslinking of about 98%. The ultrathin PA nanomembrane has excellent desalination performance beyond the existing upper limit of selective permeability and is unmatched by state-of-the-art RO membranes.

### 3.2. COF

Covalent organic frameworks (COFs) are a new class of highly porous crystalline polymer linked by strong covalent bonds, and are recognized as sister materials to MOFs [31,32,33]. COFs have been proposed as ideal candidate materials for advanced separation applications because of their inherent porosity, ordered channel structure, large surface area, and abundant hydrogen bonding sites [34,35]. Tunable pore size, ordered one-dimensional nanochannels, and easily customizable features make COFs the best candidates for building molecular sieve membranes with excellent properties [36]. COF membranes are realized by in situ growth [34], interfacial polymerization [36,37], mixed matrix membranes [38], and layer-by-layer synthesis [39].

**Figure 3:**
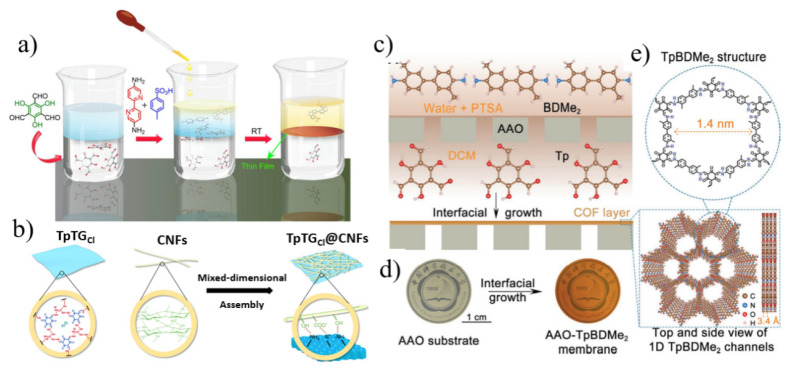
(**a**) Scheme of fabricating COF membranes by a liquid−liquid IP method. The bottom colorless layer, the blue layer, and the top yellow layer correspond to aldehyde in dichloromethane solution, water as the spacer solution, and amine-p-toluene sulfonic acid aqueous solution, respectively. (**b**) Scheme of mixed-dimensional assembly of COF nanosheets and CNFs. (**c**) Schematic of preparation of TpBDMe_2_ membranes by an interfacial growth. (**d**) Photographs of AAO substrate and AAO supported TpBDMe_2_ membranes with a diameter of ≈2 cm. (**e**) The structure of TpBDMe_2_ membranes with 1D nanochannels of 1.4 nm and hydrogen bonding sites (-NH) on the channel wall. Reprinted with permission from [40,43,44]. Copyright (2017) American Chemical Society. Copyright (2019) the author(s). Copyright (2021) Wiley-VCH GmbH.

Most conventional COF synthesis methods have poor control over their morphology and result in insoluble and unprocessable microcrystalline powders. Dey et al. [40] fabricated high-aspect-ratio COF films from COF microcrystalline powders through an interfacial crystallization strategy (Figure 3a). Using the liquid–liquid interface as a platform at room temperature, free-standing porous 2DNM membranes can be easily obtained and easily transferred to various substrates. These nanostructured COF films exhibited excellent water permeability (211 L m^−2^ h^−1^ bar^−1^) and solute rejection. Although some attempts have been made to fabricate COF mixed matrix membranes, the progress has been limited so far, among which the main problems are the poor bonding between COF thin films and porous supports and the insufficient stability of the prepared composite membranes. A huge obstacle preventing the application of COF membranes in water treatment is their insufficient hydrothermal stability. Fan’s group [41] presented a two-dimensional imine-linked COF-LZU1 membrane supported on alumina tubes by in situ solvothermal synthesis. In order to prepare defect-free and flexible COF composite molecular sieve membranes, Liu et al. [42] proposed a method to prepare COF membranes without transfer the IP method. Large-area continuous defect-free azine-linked ACOF-1 membranes were prepared by peaceful and facile in situ interfacial polymerization on hydrolyzed polyacrylonitrile (HPAN) substrates. ACOF-1/HPAN composite membrane exhibited satisfactory rejection (>90%) and an ultrahigh water permeance of 141.8 L m^−2^ h^−1^ bar^−1^ for negative dyes with molecular weight > 435 Da. The composite membrane showed remarkable stability and permeability in water and organic solvents, and good chemical stability under acidic and alkaline conditions.

Compared with graphene oxide (GO) membranes, 2D COF membranes can achieve ultrafast molecular sieving through structurally tunable and rigid frameworks. However, the pore size of chemically stable COFs is usually larger than 1 nm, which makes it difficult to accurately sieve small molecules [43]. Mixed-dimensional heterostructures can effectively control the stacking behavior of COFs, thereby regulating the structure and properties of COF films. Yang et al. [43] developed a mixed-dimensional assembly strategy to fabricate 1D cellulose nanofiber (CNF) to intercalate 2D COF nanosheet laminar flow membranes by a vacuum filtration method, as shown in Figure 3b. The shading effect of CNFs (2 nm in diameter, 0.5–1.0 µm in length) modulates the pore size (0.45–1.0 nm) of the COF nanosheets, enabling the membranes to have precise molecular sieving capabilities. In addition, the synergistic interaction between COFs and CNFs improves the mechanical strength of the film. The composite membrane exhibits excellent performance in dye rejection and desalination. In addition to this, nanoscale membranes with specific interaction sites can also be constructed by tuning the channel wall chemistry for efficient ion separation. Besides, nanoscale membranes with specific interaction sites can also be constructed by tuning the channel wall chemistry for efficient ion separation. Sheng et al. [44] constructed ultrathin nanoporous COF membranes with one-dimensional channels and abundant hydrogen bonding sites on porous substrates by interfacial growth. 1,3,5-Triformylphloroglucinol (Tp) and 2,6-dimethylbenzidine (BDMe_2_) formed a TpBDMe_2_ membrane with a pore size of about 1.4 nm at the interface of dichloromethane and water (Figure 3c–e). Hydrogen bonding interactions between hydrated cations and COF channel walls enable high ion selectivity and permeability, and divalent cations need to cross a higher energy barrier than monovalent cations when passing through COF channels.

### 3.3. Nanoporous Graphene Membranes

Graphene, the monolayer of graphite, is made up of periodically tightly packed carbon atoms in a hexagonal honeycomb structure [45,46,47]. As the thickness of a single graphene sheet corresponds to the thickness of a single carbon atom (i.e., 0.3 nm), its unique structure is simplified to 2D [48]. The unique property of graphene, its single-atom thickness, has many promising applications in a wide range of fields, especially separation science. Research has shown that pristine graphene is permeable to protons [49], but not to any molecule, even the tiniest helium atom [50]. This is because the π-orbitals in graphene lead to the formation of dense, delocalized clouds that block the gaps within its aromatic rings [51].

Graphene synthesis methods are divided into top-down and bottom-up methods. Top-down approaches to graphene synthesis include chemical, liquid-phase, mechanical, and electrochemical exfoliation. Bottom-up graphene synthesis methods include chemical vapor deposition (CVD) epitaxial growth methods, etc. The bottom-up approach to making graphene is easier to scale and less expensive than the top-down approach. The interest in graphene in membrane science stems from its properties that are stronger, thinner, and more resistant to chemical corrosion than the polyamide active layer in thin-film composite RO membranes. Grasso et al. developed a novel composite membrane (PVDF-f-G) by grafting an appropriately functionalized polyvinylidene fluoride (PVDF) membrane (PVDF-f) with graphene. When applied to direct contact membrane distillation (DCMD), PVDF-f-G membranes exhibited durable salt rejection (above 99.9%) and improved temporal stability [52]. Graphene membranes are expected to be candidates for next-generation liquid separation systems.

The carbon atomic thickness, superior mechanical strength, and chemical inertness of graphene have inspired intensive research into drilling holes in graphene nanosheets to develop nanoporous graphene (NPG) films [51]. Nanoporous graphene with high density and uniform subnanopores is expected to be an efficient molecular sieve membrane. Through classical molecular dynamics simulations in 2012, Cohen-Tanugi and Grossman elucidated that the water permeability of single-layer nanoporous graphene membranes is orders of magnitude higher than that of conventional reverse osmosis membranes [53]. Due to the size exclusion mechanism, the size of the nanopores has a great influence on its separation performance, where larger pore size leads to higher water permeability and lower rejection. Nanopores can also be functionalized with hydrogen and hydroxyl groups to further enhance the selectivity of NPG through charge repulsion [53,54]. Subsequently, the desalination performance of graphene films with pores of 14.5, 10.5, 7.5 Å with different functional groups was investigated [55]. The results showed that the ionic free energy barrier and desalination performance were affected by the pore diameter. Under electrostatic and steric effects, the free energy barrier of ions can be increased by the functionalization of charged groups. Although the theoretical basis has been established [56,57], creating nanopores with uniform pore size and high pore density remains a formidable challenge. The drilling techniques currently used to fabricate nanoporous graphene include ion bombardment [58], oxygen/hydrogen/argon plasma etching [59,60,61], ion beam irradiation, thermal annealing [62,63], etc. Electron beam irradiation can produce well-defined pore sizes, but cannot achieve efficient molecular separation due to its large pore size from 3.5 to 100 nm. Oxidative etching, ion bombardment or plasma etching can be precisely controlled and create sub-nanopores.

**Figure 4:**
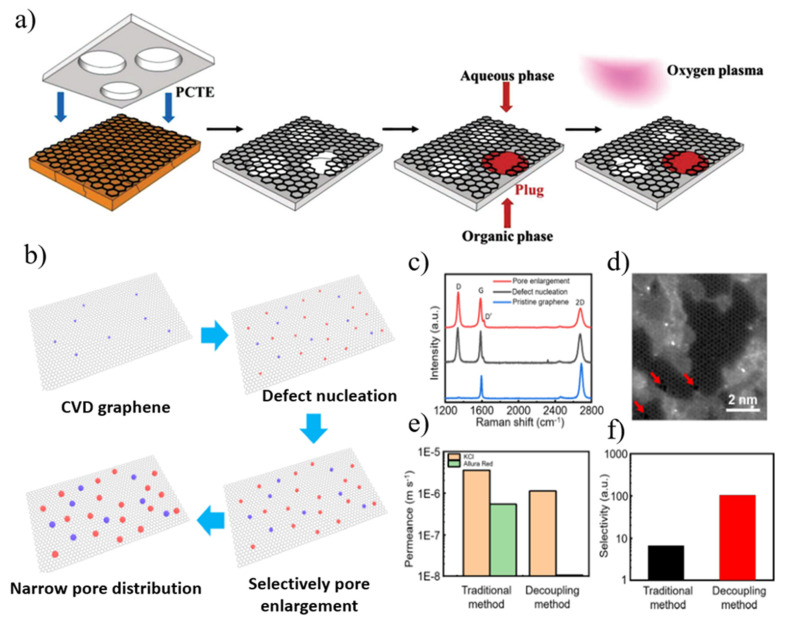
(**a**) Synthesis of nanoporous atomically thin membranes (NATMs). Schematic overview of the process to synthesize large-area NATMs from CVD graphene grown on Cu foil. (**b**) Decoupling method involving CVD and two-step plasma treatment for the fabrication of atomically thin nanoporous graphene. (**c**) Comparison of Raman spectra of pristine graphene, defect-nucleated graphene, and pore-enlarged graphene. (**d**) Atomically resolved image of treated graphene. The nanopores are marked using red arrows for better illustration. (**e**) Permeance of KCl and Allura Red of NATMs prepared using the traditional method and the decoupling method. (**f**) Calculated selectivity of KCl to Allura Red based on the permeance data in (**e**). Reprinted with permission from [59,60]. Copyright (2017) WILEY-VCH Verlag GmbH & Co. KGaA, Weinheim. Copyright (2021) American Chemical Society.

The chemical vapor deposition (CVD) method is currently an effective method to prepare high-quality graphene films in large areas. However, CVD-grown graphene films will produce defects, grain boundaries, and wrinkles during the preparation process, and surface contamination and damage will also occur during the transfer process, thus limiting further applications. In some studies [60,64], interfacial polymerization has been adapted to effectively plug leaks in graphene tears/damages. Kidambi et al. [60] fabricated square centimeter-scale nanoporous atomically thin films (NATM) by transferring CVD-grown graphene onto a polycarbonate orbital etch (PCTE) support followed by interfacial polymerization to seal defects and oxygen plasma etching to generate selective pores (Figure 4a). Permeability was increased by about 1–2 orders of magnitude compared to the polymer membranes of the time, which were formed using a simple and scalable process for desalination, dye rejection, and small molecule separation.

The simultaneous introduction of pore generation and enlargement in conventional etching methods often results in a trade-off between pore density and pore size distribution. Recently, Chen and his colleagues [59] used a two-step plasma irradiation technique to prepare porous graphene with tunable pore density and pore size (from sub-nanometer to several nanometers). Defects in graphene are first created by etching with low-energy argon plasma, and then by using controlled oxygen plasma to tune the desired nanopore size (Figure 4b–f). The pore density can be effectively tuned by varying the number of bombarding argon ions, and the nanopore size increases with the oxygen plasma treatment time. The as-prepared nanoporous graphene membrane with a surface area of 1 cm^2^ had a selectivity value of 104 for KCl and Allura Red and a permeability of 1.1 × 10^−6^ m s^−1^. Notably, they used interfacial polymerization to seal the graphene cracks and tears prior to the two-step plasma treatment.

### 3.4. Others Nanoporous Membrane

Inspired by nanoporous graphene, the potential of other 2D nanosheet membranes has also been exploited. In 2015, Heiranian et al. [65] showed through MD simulations that drilling holes in MoS_2_ could achieve 70% greater flux than nanoporous graphene, and that pore chemistry plays an important role in regulating water flux. The potential advantage of fabricating large-area high-quality monolayers makes MoS_2_ a promising candidate material for the development of novel 2D nanosheet films [66]. Furthermore, nanoporous BN has higher flux than graphene with the same pore size. Four functionalized nanoporous BN (BNNS) nanosheet membranes including hydrogen (-H) and fluorine (-F) were designed by Jafarzadeh et al. [67]. According to DFT and MD simulations, the BNNS membrane could completely remove Na^+^ and Cl^−^ ions from water at low pressure (*p* < 50 MPa). Two-dimensional polymer membranes with high-density uniform-sized nanopores were fabricated by a bottom-up approach, which also exhibited excellent ion entrapment and ultra-high water flux [68]. However, these studies are all based on theory and have not been widely used.

## 4. 2D Lamellar Membranes (Layered Stacks)

Apart from the use of 2D nanoporous sheets, 2DMs can be readily assembled into laminar layers with a well-defined interlayer distance which can act as a separation channel. Several outstanding studies are reported in the following section that use these 2D layered stacks for designing high-performance membranes.

### 4.1. Graphene Oxide Membranes

Graphene oxide (GO), an oxidized form of graphene, is an indispensable component of two-dimensional separation membranes [51,69]. The structure of GO nanosheets is a single-atom-thick layer with a lateral dimension reaching tens of micrometers. GO nanosheets can be mass-produced by the chemical oxidation of graphite and ultrasonic-assisted rapid exfoliation [46]. Moreover, the oxygen-containing functional groups of GO nanosheets endow it with excellent processability, allowing it to be assembled into ordered structures with nano- or even sub-nanometer-sized channels [70]. In fact, stacking and arranging GO nanosheets into layered membranes has been recognized as a scalable and cost-effective method for water treatment. In particular, the oxygen-containing groups of GO provide active sites for further functionalization to enhance properties such as charges and specific interactions with ions and molecules [46]. In addition, persistent interlayer hydrogen bonds hold the GO sheets together to form stable freestanding films [71].

Various techniques such as drop casting [72], spray coating [73], dip coating [74], layer-by-layer (LBL) assembly [75,76], and vacuum/pressure [77,78] have been used for the fabrication of graphene- and composite-based nanofiltration membranes. Drop casting is a simple method to fabricate GO films that is insensitive to the shape of the substrate. However, a major disadvantage of this technique is that even under almost ideal conditions, concentration gradients in the liquid phase or differences in evaporation rates across the substrate can lead to changes in the thickness or internal structure of the entire film. Spin coating is a fast, direct and simple process for depositing GO layered films, but with low film strength. The LBL strategy is able to control GO laminate thickness by managing the deposition cycle. Vacuum and pressure-assisted filtration methods are the most common and straightforward methods for the large-scale fabrication of free-standing GO-based nanofiltration, microfiltration, and ultrafiltration membranes. Generally, the thickness of the prepared membrane depends on the concentration and volume of the GO suspension in the vacuum filtration system unit. Due to the pressure difference between the bottom and top membranes, the prepared membranes may have disordered morphologies and different layer arrangements in thicker membranes, leading to more disordered morphologies [79].

The selective permeability of GO nanosheets is controlled by three transport channels: interlayer channels formed by face-to-face nanosheet interactions, structural defects, and slit-like pores formed by edge-to-edge nanosheet interactions [51,80]. In GO lamellar membranes, molecules first pass through slit-like nanopores and then smoothly permeate through interlayer channels. For the first time, Nair et al. [81] found that GO-based membranes can completely repel liquid and gas molecules and only allow water molecules under dry conditions. Since oxygen-containing functional groups tend to aggregate, they can cause wrinkling of the sheets, resulting in waviness. This behavior results in the formation of large permeable regions in non-graphene oxide. When the GO membrane is immersed in water, hydration increases its d-spacing, allowing high permeability for small molecules smaller than 0.45 nm in size, while blocking larger species [82]. Numerous studies have shown that interlayer channels between GO nanosheets play a crucial role in the fast and selective transport of water, ions, and gases.

In addition, the swelling of GO laminates in water adversely affects the repulsion of ions and desalination must be prevented [83]. Conclusively, the oxygen-containing functional groups on the GO sheets are ionized in the wet state and thus are negatively charged. This fact affects the mobility of anions and cations. Therefore, the selectivity of GO multilayers is determined by the size exclusion effect depending on the GO sheet spacing, the electrostatic interaction between ions and negatively charged GO sheets, and the ion adsorption effect [71].

**Figure 5:**
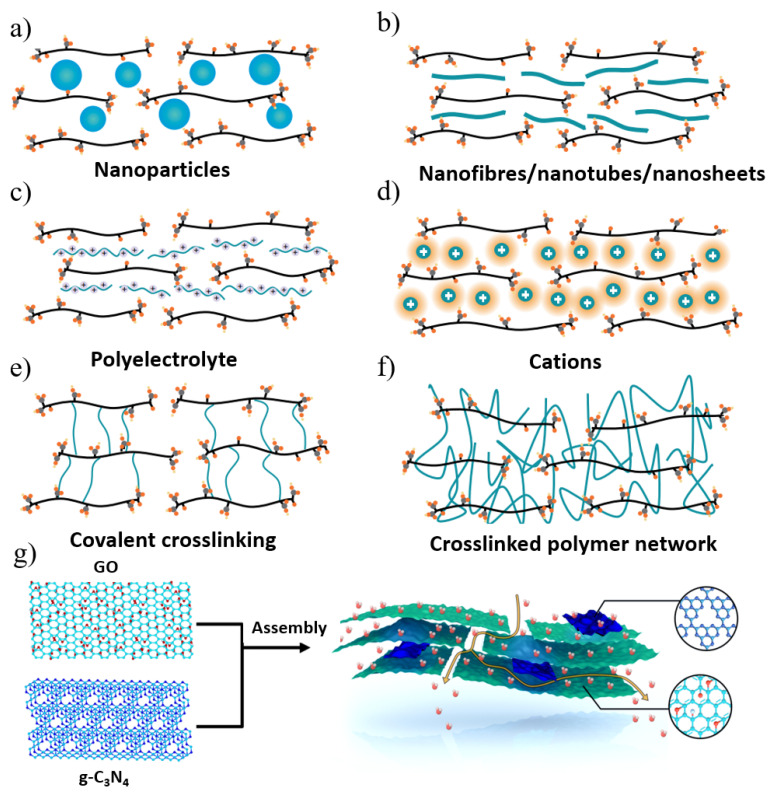
Main strategies for the stabilization of GO membranes and the control of interlayer spacings and their mass transport. (**a**) Nanoparticles; (**b**) nanofibres/nanotubes/nanosheets; (**c**) polyelectrolyte; (**d**) cations; (**e**) covalent crosslinking; (**f**) crosslinked polymer network. (**g**) Illustration of the assembly of GO and g-C_3_N_4_ sheets and the transfer mass process. Reprinted with permission from [86,87]. Copyright (2021) Springer Nature Limited part of Springer Nature. Copyright (2021) American Chemical Society.

GO membranes tend to swell when immersed in aqueous solutions, limiting the potential for ion filtration applications; thus, reducing the interlayer spacing sufficiently to exclude small ions is a challenge. The above problems are solved by controlling the stacking of GO nanosheets to form ordered interlayer channels to achieve ideal water–solute separation. As shown in Figure 5a–f, many physical and chemical methods have been employed to fabricate GO-based membranes to obtain stable structures, diverse surface functions, and excellent separation properties. These strategies effectively tune the interlayer spacing of GO sheets without changing the density and distribution of surface functional groups to ensure favorable water–GO interactions.

Since GO sheets are rich in abundant oxygen-containing functional groups which make GO easily exfoliated in solution, they can also be used to induce other chemical reactions to generate additional functional groups. For example, these additional groups can allow GO layers to intercalate or crosslink with primary complex monomers, or covalently link to polymers. These properties have been used to provide a controlled way to reduce the interlayer spacing of layered GO films, which also contributes to their overall stability due to covalent bonding [82]. Guo and co-workers [84] embedded amino-functional crosslinkers (EDA and PEI) of different sizes into GO nanosheets to achieve GO network membranes with ultrafast permeability and excellent durability. Studies have shown that the different bridging abilities and steric hindrances produced by intercalators of different sizes have different effects on GO stacking. Small molecules contribute to in-plane orientation, while large-sized crosslinkers inhibit the aligned stacking of adjacent GO sheets resulting in lower degrees of alignment, shorter transmembrane pathways, and ultimately higher permeability. Xu et al. [85] developed Fe/GO-TAx membranes obtained from tannic acid-functionalized GO (GO-TA) nanosheets coordinated with Fe^III^ ions. Fe/GO-TAx films show increased interlayer distance, reduced roughness, stronger stability, and enhanced permeability compared to pristine GO films. Therefore, crosslinking is crucial to maintain the stability of GO layered films.

**Figure 6:**
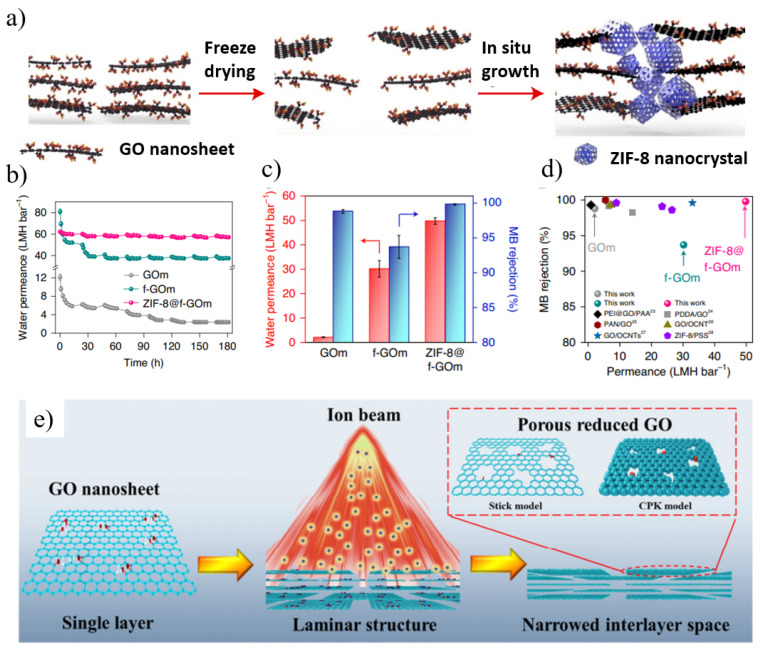
(**a**) Schematic of ZIF-8@f-GOm preparation. (**b**) Pure water permeance (in LMH bar^−1^) of GOm, f-GOm and ZIF-8@f-GOm over a 180 h operating period (**c**) Water permeance and MB rejection for GO-based membranes. (**d**) Comparison of MB rejection and water permeance for GO-based membranes reported in the literature with the GO-based membranes used in this work. (**e**) Schematic illustrations of ion beam engineering for GO membranes. Reprinted with permission from [90,91]. Copyright (2021) the author(s), under exclusive license to Springer Nature Limited. Copyright (2019) Published by Elsevier Ltd.

In order to improve the permeability and selectivity of GO membranes, other suitable strategies were also employed to minimize the hydration effect, thereby improving the separation performance of small molecules while improving the stability of the membrane [86]. Wu’s group [87] successfully constructed GO/g-C_3_N_4_ films by adding g-C_3_N_4_ sheets in the GO interlayer by a simple hybrid method (Figure 5g). Due to the ultralow friction water flow of g-C_3_N_4_ nanosheets, the permeation flux of the composite membrane was greatly enhanced. The GO/g-C_3_N_4_ membrane exhibited good long-term stability, antioxidation and acid-base resistance. Luo et al. [88] fabricated an attapulgite (ATP) nanofiber/GO (ATP/GO) composite membrane by embedding ATP nanofibers into a sandwich of stacked GO nanosheets. By adding ATP, the gap of the GO layer in the membrane was significantly increased, thereby expanding the mass transfer nanochannels and enhancing the permeation of water through the membrane (221.16 L m^−2^ h^−1^ bar^−1^), which was more than seven times higher than that of the pure GO membrane. At the same time, the layered ATP/GO membrane exhibited high dye rejection not inferior to the GO membrane. Sun and his colleagues [89] fabricated GO-PVA films by intercalating and crosslinking polyvinyl alcohol (PVA) to efficiently tune the interlayer structure. The resulting crosslinked GO membranes exhibited excellent water production, high salt rejection, and extraordinary stability. Zhang et al. [90] reported a new strategy for the in situ crystallization of zeolite imidazolate framework 8 (ZIF8) nanocrystals along the edges of GO nanosheets (Figure 6a–d). The tailored growth of ZIF-8 nanocrystals on GO microporous defects made the framework structure stable under cross-flow conditions and applied hydraulic pressure, even with only a small decrease in water permeability after lyophilization. ZIF-8 nanocrystals not only improved the framework stability but also increased the selectivity of water–solute separation, enabling ZIF-8@f-GOm to achieve ultra-high permeability and long-term stability (180 h). The precise filling of microporous defects of GO-based films with ZIF-8 provides new insights into designing 2D laminated films with high mechanical stability.

The performance of GO membranes is limited by permeation through long and tortuous diffusion paths, resulting in lower water permeability [91]. Mi [92] proposed a promising strategy to fabricate stacked nanoporous graphene (SNG) membranes from nanosheets in a similar way to fabricating GO membranes. When stacking several layers of nanoporous graphene sheets, the functional groups at the edges of the nanopores will act as spacers to keep the interlayer channels open [92]. Compared with nanoporous single-layer graphene films and stacked GO films, SNG films should have better scalability and higher selectivity. Cohen-Tanugi et al. [93] utilized classical molecular dynamics simulations to evaluate the potential and design of multilayer NPG membranes via bilayer NPG. Smaller layer separation with perfectly aligned pores can not only significantly facilitate water permeation but also enhance the salt rejection of the membrane, while larger pore offset can significantly facilitate salt rejection. Kang et al. [94] activated GO at 650 °C to prepare NPG, which was then treated with KMnO_4_ to graft oxygen-containing functional groups, and finally vacuum-filtered to form a functionalized nanoporous graphene (FNG) membrane. The FNG membrane remains stable under cross-flow filtration as it retains high water flux (586 L m^−2^ h^−1^ bar^−1^) and excellent molecular separation (molecular weight cut off: 269 Da). Wei et al. [91] proposed a facile and rapid ion beam-induced preparation of ion-selective GO membranes, which is an effective method for simultaneous in situ reduction and defect engineering (Figure 6e). The layered structure of GO films can be controllably tuned in reducing interlayer space and tailoring nanoscale pores. Compared with the ion separation performance of GO membranes without ion beam treatment, the ion selectivity of GO membranes irradiated with ion beams is enhanced.

Some attempts have been made to incorporate GO nanosheets into a polymer material in order to fabricate the overall high performance of mixed matrix membranes (MMMs). The main advantage of the mixed matrix strategy is the facile combination of the excellent processing ability of polymers and the unique characteristics of the 2D materials such as thermal stability, high stiffness, and other specialties. However, a relatively poor compatibility/affinity between GO (inorganic filler) and soft polymer inevitably results in nonselective interfacial defects and GO aggregates, potentially impairing the separation and mechanical properties of GO-modified membranes. To address this issue, GO sheets can be functionalized with organic groups to enable favorable compatibility with organic polymer chains. In one example, surface zwitterionization of GO was conducted by grafting poly (sulfobetaine methacrylate) (PSBMA) onto the GO surface, followed by blending with polyethersulfone (PES) to prepare a novel loose NF membrane. The resulting hybrid membrane exhibited substantially improved water permeability, a high rejection of reactive dyes, and excellent antifouling performance [95].

GO membranes have been widely used in water treatment, but they are unstable in an aqueous environment due to the hydrophilicity of oxygen-containing functional groups. Ensuring the stability of the membrane by crosslinking or intercalation inevitably increases the structural complexity and heterostructure of the membrane. Its water permeability and water repellency properties are hardly comparable to those of polymer desalination membranes, and more extensive and in-depth research is needed.

### 4.2. RGO Membrane

Another derivative of graphene, reduced graphene oxide (rGO), can restore its graphene-like properties by reducing GO [96]. The residual oxidized functional groups and defects on the rGO sheets generated during the oxidation/reduction process enhance the processability of rGO and facilitate its functionalization and modification [97]. Furthermore, the *π–π* interaction between the GO sheets is enhanced by the reduction process, which improves the stability of the GO film. The reduction of GO films with hydrothermal treatment [98], thermal annealing [99], electron beam irradiation [100], laser-reduced [101] or chemical reduction (including hydrazine, ascorbic acid, etc.) can remove oxygen-containing functional groups. Therefore, this process narrows the interlayer space between the GO sheets and increases the barrier properties of the film [71]. The degree of reduction is also the key to the separation performance, so it is necessary to adjust the temperature, the ratio of GO to reducing agent, the sonication time, power and pH value, etc. [51,101].

**Figure 7:**
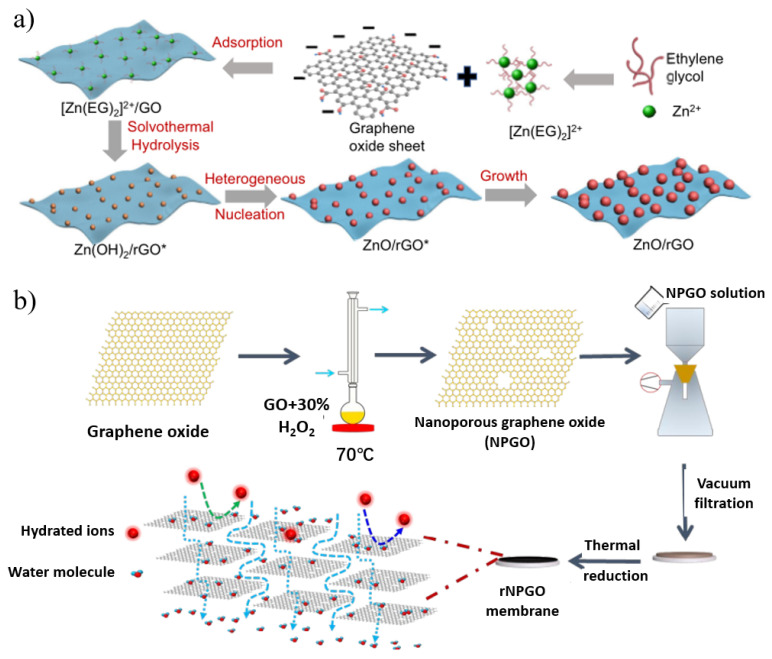
(**a**) Schematic illustration of the growth mechanism of ultrafine ZnO/rGO nanocomposites. (**b**) Schematic illustration of the fabrication process of reduced nanoporous graphene oxide (rNPGO) membrane. Reprinted with permission from [106,107]. Copyright (2019) American Chemical Society. Copyright (2022) published by the author(s).

Reduced graphene oxide (rGO) membranes with lower oxygen functional groups have better molecular sieving ability because of their narrower nanochannels, lower swelling, and greater stability in water [102]. Unfortunately, water permeation through the stacked GO membranes is insufficient due to strong capillary forces and narrow nanochannels. Widening the interlayer spacing has been used to modulate permeation in rGO films, and can comprise cationic crosslinking [103], carbon nanotube intercalation [104], polymer additives [105], etc. Zhang et al. [106] demonstrated a versatile and facile method to synthesize ultrafine metal oxide/rGO nanocomposites through a heterogeneous nucleation and growth process for nanofiltration applications (Figure 7a). The uniform adhesion of these ultrafine metal oxide nanoparticles effectively suppressed the wrinkling and stacking of the resulting rGO sheets. By acting as pillars, the nanoparticles significantly increased the vertical interlayer spacing and lateral tortuous path of the rGO film, and water molecules could easily pass through the narrow spaces between the nanoparticles, while macromolecular dyes were selectively rejected. Compared with traditional nanofiltration membranes, ultrafine metal oxide/rGO membranes address the key trade-off between water flux and dye rejection.

However, the removal of oxygen-containing functional groups on the surface of rGO nanosheets generally results in lower water permeability and narrow interlayer spacing after the reduction process due to the reduced hydrophilicity of the membrane [107,108,109]. To address this problem, one solution is to introduce in-plane nanopores to make rGO nanosheets porous. For example, Li’s group [107] fabricated reduced nanoporous GO (rNPGO) membranes by the vacuum filtration of nanoporous graphene followed by heat treatment (Figure 7b). Its water permeability is 26 times higher than that of non-porous rGO prepared under the same conditions because the in-plane nanopores provide additional transport channels and shorten the transport distance of water molecules. On the other hand, GO flakes can be selectively reduced to have low epoxy content and high hydroxyl content. The hydrophilic hydroxyl groups and aromatic rings form a low-friction surface that can facilitate water permeation through the GO membrane. While the epoxy groups contribute little to the formation of hydrogen bonds during GO dissolution, it may increase the mass transfer resistance to reduce permeation. Yi et al. [110] used different ratios of isopropanol-modified EBI-selective reduction to prepare GO membranes with ultra-high water permeability. The EBI-rGO membrane meanwhile still maintains the efficient rejection of dyes (MB, PR, RB) and polyvalent metal ions (Fe^3+^, Pb^2+^, Cu^2+^) with a flux as high as 267 L m^−2^ h^−1^ bar^−1^. The hydrogen radical (H•) generated by EBI reduces the epoxy group to generate a new hydroxyl group which plays an important role in the selective reduction process.

### 4.3. MXene Membranes

MXenes, a general term for transition metal carbon/nitrides, are a new class of two-dimensional nanomaterials developed in recent years [111]. Due to their high aspect ratio, abundant surface groups, good electrical conductivity, and natural hydrophilicity, they have been widely used in energy storage, catalysis, sensing, and the environment [112,113]. MXenes are regarded as promising candidate materials in the field of membrane separation, and the most widely used MXene is Ti_3_C_2_T_x_ (T = O, F, OH) [114]. The fast transport of molecules within membranes relies on the orderly flow of molecules in interlayer channels and pores between nanosheets [115]. Compared with other sheet materials, MXenes have better rigidity, abundant surface groups, and natural hydrophilicity, so MXenes have great potential to construct high-performance separation membranes.

**Figure 8:**
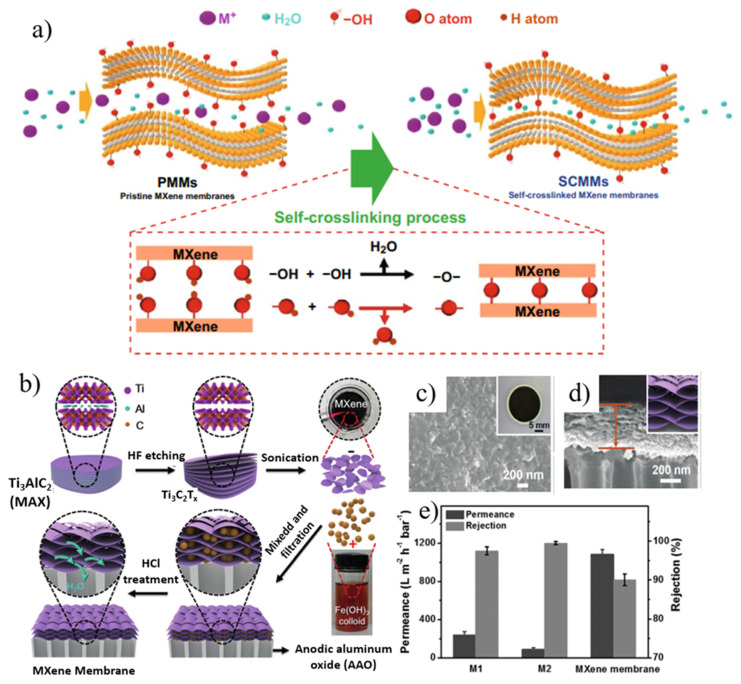
(**a**) Illustration of the self-crosslinking process. Reproduced with permission. (**b**) MXene membrane preparation. (**c**) SEM image (inset: macroscopic photograph) of the MXene membrane surface. (**d**) High magnification of SEM image of the cross-sectional view of the MXene membrane (inset: representation of the layered structure). (**e**) performance of MXene-based NLMs with enlarged interlayer spacing achieved by the intercalation and subsequent removal of colloidal Fe(OH)_3_. Reprinted with permission from [117,121]. Copyright (2019) Published by American Chemical Society. Copyright (2017) Published by Wiley-VCH Verlag GmbH & Co. KGaA, Weinheim.

The first MXene-based separation membrane was fabricated by Ren et al. [116] in 2015, and was a pioneering achievement in the membrane field. The self-supporting membrane was prepared by the VAF method with a nanochannel diameter of 0.63 nm. It enabled a highly selective screening of cations and exhibited better selectivity than GO for multivalent ions. However, MXenes suffer from the problem that water molecules and ions tend to intercalate between adjacent interlayers in water, resulting in swelling. This problem can be effectively solved by constructing an interlayer crosslinking structure which can be adopted in two ways: (1) a self-crosslinking strategy; (2) an ionic crosslinking structure. Through a simple heat treatment, a self-crosslinking MXene film with anti-swelling was synthesized by utilizing the functional groups on MXene nanosheets (Figure 8a) [117]. The Ti-O-Ti bonds formed by dehydroxylation between adjacent interlayers can stabilize the interlayer space, and the swelling of MXene films is significantly inhibited by self-crosslinking. After crosslinking, the leakage rate of metal ions is reduced by an order of magnitude, so the material can be stably used for metal ion screening. As expected, the self-crosslinked MXene membrane exhibited significant swelling inhibition and excellent monovalent ion repulsion during long-term ion separation for 70 h with good long-term stability. In another work, Ding et al. [118] formed ionic bonds between MXene sheets through Al^3+^ ion intercalation, and the enhanced interlayer stable structure effectively suppressed the swelling of MXene films in aqueous solution. After a long-term ion test, the Al^3+^ in the crosslinked film was not lost from the interlayer, and it had good structural stability. These membranes exhibited excellent non-swelling stability over 400 h of continuous operation with high NaCl rejection (96.5%) and fast water flux (2.8 L m^−2^ h^−1^ bar^−1^). In addition, combining MXenes with other existing technology-proven materials (e.g., polymers [119], carbon nanotubes [120], etc.) to make composite membranes also can relieve swelling.

However, the size of the interlayer transport channel formed by the close and free stacking of MXene 2D nanosheets is limited, which affects the transfer rate of water molecules in the membrane channel, thereby limiting the improvement of membrane permeation flux. Ding and his colleagues [121] used nanoparticle intercalation to expand the interlayer channel size, thereby enhancing membrane permeation flux (Figure 8b–e). Positively charged Fe(OH)_3_ colloids intercalated into negatively charged MXene nanosheets by electrostatic force, forming extended nanochannels, increasing the volume of interlayer cavity, and improving membrane permeability. The layered MXene membrane achieved a water flux of over 1000 L m^−2^ h^−1^ bar^−1^ and a high rejection (90%) for molecules larger than 2.5 nm. Recently, Liao’s group [122] prepared ultrathin zwitterionic MXene (Z-MXene) nanosheet-intercalated GO nanomembranes with size-selective permeability by a slow deposition method. The dual-functionalized Z-MXene acted as a directional intercalator, preventing the self-weight stacking phenomenon and obtaining multicomponent layered nanofilms with dual-ordered nanochannels within a finite spacing. As expected, the flux of the composite membrane achieved a high leap from 32.1 L m^−2^ h^−1^ bar^−1^ to 115.5 L m^−2^ h^−1^ bar^−1^, with a satisfactory rejection (99.1%) for small molecules, surpassing most GO-based 2D/2D layered films and GO-based ultrathin films. Furthermore, additional holes can be drilled in initially defect-free MXene nanosheets, further enhancing the obtained permeability. Nanoporous Ti_3_C_2_T_x_ MXene layered structured films were obtained by partial etching with weak acid oxidant H_2_SO_4_. The use of a mild acid oxidant neither altered the crystallinity nor affected the surface functionality of the unetched portion of the Ti_3_C_2_T_x_ sheet. The etched pores acted as interconnected cation channels and thus the nanoporous MXene layered membrane enhanced permeability and selectivity with this method.

**Figure 9:**
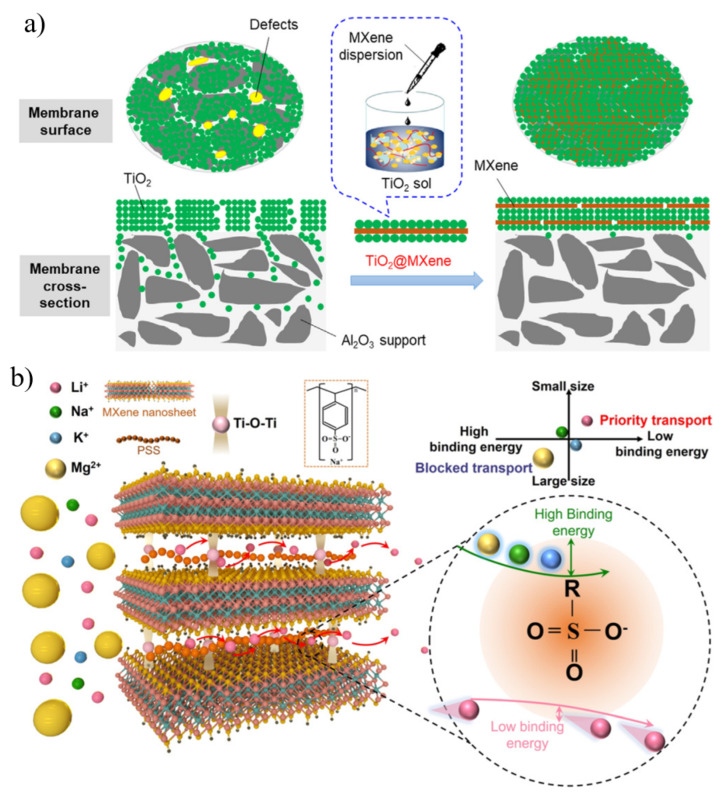
(**a**) Fabrication of TiO_2_-MXene membranes to eliminate potential defects. (**b**) Diagrammatic sketch of the fast transport subnanochannels for Li^+^ in MXene/PSS composite membranes. Reprinted with permission from [124,125]. Copyright (2018) Published by Elsevier B.V. Copyright (2021) Published by Wiley-VCH GmbH.

The disordered stacking of nanosheets in conventional 2D membranes is prone to defects, leading to low selectivity. Mixing Ti_3_C_2_T_x_ with other nanomaterials (such as GO) to form a film and using the complementary relationship between the structures and sizes of the two materials can also achieve the purpose of regulating the interlayer channel and improving the separation performance of the membrane. Kang et al. [123] greatly improved the dye-rejection properties of MXene-based films by incorporating GO into Ti_3_C_2_T_x_ films. During the pressure-driven filtration experiments, the MXene–GO composite membrane exhibited high rejections for methylene blue (MB) and brilliant blue (BB) with a hydration radius greater than 5 Å, but dye molecules with smaller water values and the same negative charge showed poor retention. In addition to mixing GO with MXene, TiO_2_ nanoparticles were also introduced to seal latent defects and improve selectivity. Xu et al. [124] prepared mesoporous TiO_2_-MXene films by assembling two-dimensional MXene nanosheets and TiO_2_ nanoparticles on macroporous supports (Figure 9a). Since MXene acts as a “floor tile” to block very large defects and prevent TiO_2_ gel penetration, the prepared membrane provides better glucan rejection without significantly sacrificing water flux. In addition, the selective screening of ions can also be achieved by chemically modifying confined channels. Lu and his partners [125] proposed that two-dimensional (2D) layered membranes with Li^+^ selective transport channels could be used for lithium extraction from salt lakes (Figure 9b). Poly (sodium 4-styrene sulfonate) (PSS) with active sulfonate sites was introduced into stacked MXene nanosheets to construct MXene@PSS composite membranes for Li^+^ separation. Through self-crosslinking and chemical modification, the permeability of Li^+^ decreased, but its selectivity increased by an order of magnitude. Compared with 2D membranes and polymer material membrane systems, MXene@PSS composite membranes have competitive advantages.

In addition to outstanding permeability and ion selectivity, MXene membranes demonstrate superb light-to-heat conversion efficiency, making them ideal applicants for application in solar-driven distillation. The traditional membrane distillation process is a thermally driven process in which water vapor rather than liquid water crosses the membrane, promoted by the vapor pressure difference. In most solar-driven distillation process, the MXene membrane is placed on top of the desalination device, acting as the heat absorber and vapor evaporator in which the water infiltrates the membrane through the capillary effect. A delicately designed photothermal evaporation system was developed by employing a polydimethylsiloxane (PDMS)-modified self-floating Ti_3_C_2_ MXene thin membrane through loading on porous and robust PVDF substrates, achieving a 100% internal light-to-heat conversion efficiency and 74% light-to-water evaporation efficiency under one sun irradiation [126].

Since membranes are inevitably plagued by fouling (bacteria and oil) and frequent membrane replacement is neither practical nor economical, it is necessary to develop antifouling membranes. Rasool et al. [127] first found that the membrane of MXene can inhibit bacterial growth, and then Pandey et al. [128] introduced Ag nanoparticles (AgNPs) with different loadings (between 0% and 35%) into MXene by self-reducing silver nitrate on Ti_3_C_2_T_x_ nanosheets. Compared with pristine MXene membrane (about 118 L m^−2^ h^−1^ bar^−1^), 21% Ag@MXene exhibited excellent performance in both water flux (420 L m^−2^ h^−1^ bar^−1^) and bacterial inhibition rate (Over 99% inhibition against *E. coli*). The significant increase in permeability may be due to the formation of new nanopores in the membrane by the attached silver nanoparticles, while the enhanced inhibition rate is caused by the synergistic antibacterial effect of AgNPs and MXene. A new type of promising layered bismuth-based semiconductor material, similar to Bi_2_O_2_CO_3_, BiOCl, and Bi_2_WO_6_, has been demonstrated with excellent activity, stability, and light response to the visible region, and has thus drawn the attention of recent researchers. These bismuth photocatalysts have mostly been mostly explored by being blending with existing 2D membrane materials, such as MXene and GO, which not only improves permeability but also promotes the photocatalytic activity of the composite membrane. They can be effectively used for dye degradation, antibiotic removal, inhibiting bacterial growth, and solving membrane descaling problems [129].

Although MXene membranes have excellent separation effects, they are prone to oxidation and degradation in humid environments [114]. Various studies have been carried out to find suitable preservation methods for MXenes and enhance their antioxidant capacity [130,131]. Recently, Gogotsi’s team [132] made a major breakthrough in improving the stability of MXene. They added excess aluminum in the preparation of the MAX precursor, which significantly improved the shelf-life and stability of the MXene. The oxidative stability of MXene in air was also significantly improved and could be stored in airtight vials at room temperature for 10 months. Although the water flux of the MXene intercalation membranes reported so far is greatly improved, they still cannot effectively intercept small-sized molecules. MXene two-dimensional material membranes still have problems that need to be solved, such as low separation accuracy, relatively single separation function, unclear separation mechanisms, and easy swelling in water.

### 4.4. Transition Metal Dichalcogenide (TMD) Membranes

Two-dimensional transition metal dichalcogenides (TMDs) are relatively new emerging membrane materials compared to graphene, and are composed of transition metals (Mo, W, etc.) combined with chalcogenides (S, Se, etc.) [133]. Monolayer TMD nanosheets are thicker than graphene and consist of three atoms: a layer of transition metal atoms sandwiched between two layers of chalcogen atoms [133]. Atoms in the same layer are bound by covalent bonds, and atoms in different layers are attracted by weak van der Waals bonds. TMD nanosheets can be directly obtained from TMD powders by the chemical exfoliation method using intercalators such as alkyllithium [51].

**Figure 10:**
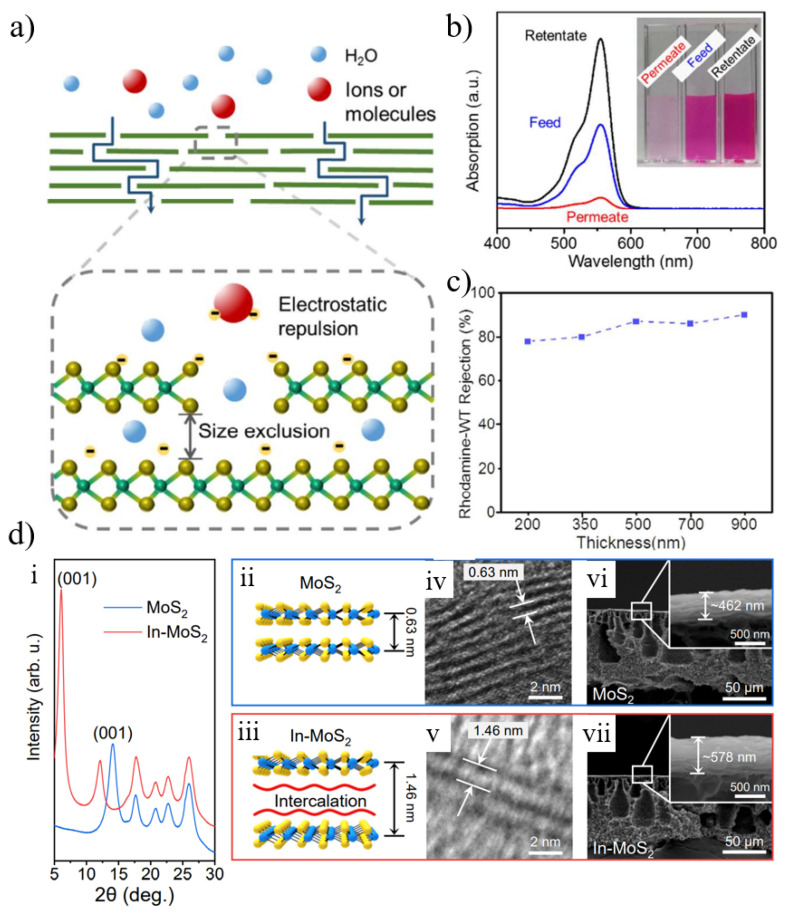
(**a**) The proposed mechanisms of MoS_2_ membranes include both size exclusion and electrostatic repulsion. (**b**) The concentrations of organic dye (rhodamine-WT) in permeate, feed, and retentate, as evidenced by the absorption spectra. (**c**) Dependency of organic dye rejection on MoS_2_ membrane thickness. (**d**) (**i**) XRD patterns of pristine and intercalated MoS_2_ membranes. (**ii**,**iii**) Schematic illustration. (**iv**,**v**) TEM image. (**vi**,**vii**) SEM cross-section images of pristine and intercalated MoS_2_ membranes. Reprinted with permission from [135,136]. Copyright (2017) Published by American Chemical Society. Copyright (2020) Published by American Chemical Society.

Among different TMD nanosheets, layered membranes reconstructed from molybdenum disulfide (MoS_2_) nanosheets have recently shown great promise in water purification and desalination due to their high water flux, excellent salt rejection, and long-term stability [134]. MoS_2_ nanosheets do not have any hydrophilic functional groups on the surface, and the stacked MoS_2_ nanosheets can be prevented from dispersing in water by van der Waals interaction. Compared to other nanomaterials, MoS_2_ nanosheets are much more rigid due to the presence of three atomic layers [134]. Due to its high surface smoothness, MoS_2_ nanosheets may result in low water pressure resistance. MoS_2_ nanosheets have a stable 1.2 nm interlayer spacing (or 0.9 nm free spacing) for hydrated nanochannels, whereas dried nanosheets have a 0.62 nm interlayer spacing (or 0.3 nm free spacing) with impermeable behavior [135]. Wang et al. [135] confirmed the negligible physical adsorption of negatively charged rhodamine-WT by examining the UV-vis spectra of the permeate and retentate (Figure 10b) and rejections at different film thicknesses (Figure 10c). They believed that the overall separation mechanism for the separation of organic dyes by MoS_2_ membranes included size exclusion and electrostatic repulsion (Figure 10a).

To achieve high selectivity and permeability for ions, the interlayer spacing needs to be tunable and controlled. MoS_2_ can be modified according to the physicochemical properties of nanomaterials and film preparation parameters to control the structure and properties of the films. Materials with special properties, such as amphiphilic molecules and nanoparticles, can be added during membrane fabrication to tune the interlayer distance. Lu et al. [136] tuned the size of the nanochannels by intercalating amphiphilic molecules, and the characteristic first-order diffraction peak of the pristine MoS_2_ film after intercalation shifted from 14.1° to 6.1° of the In-MoS_2_ film, corresponding to the enlargement of the d-spacing from 0.63 nm to 1.46 nm (Figure 10d). It was experimentally found that the thickness of the MoS_2_ film was much lower than the theoretical value, and the imperfect stacking of MoS_2_ nanosheets was the culprit for the poor separation performance. Furthermore, to improve selectivity, another strategy is to functionalize MoS_2_ membranes. For example, Hirunpinyopas et al. [137] reported that MoS_2_ membranes could be functionalized by prolonged soaking in dyes of different charges. The functionalized MoS_2_ membrane effectively intercepted 99% of seawater and exhibited excellent long-term stability (immersion in water for up to 6 months). In addition, MoS_2_ nanosheets or pre-functionalized MoS_2_ can be utilized to improve the performance of polymer films [138,139,140].

### 4.5. g-C_3_N_4_ Membrane

Graphitic carbon nitride (g-C_3_N_4_) with a graphene-like structure consists of a two-dimensional layered structure with adjacent layers held together by weak van der Waals interactions [141]. The basic structural unit of g-C_3_N_4_ is generally believed to exist in two forms: s-triazine (C_3_N_3_) and tri-s-triazine (C_6_N_7_). Among them, tri-s-triazine-based g-C_3_N_4_ is considered to be the most stable form of g-C_3_N_4_ with regularly distributed triangular nanopores (3.11 Å) throughout the layered structure [142]. Unlike the non-porous nature of MoS_2_ nanosheets, g-C_3_N_4_ has periodic ultramicropores in the plane, allowing small molecules (e.g., H_2_, water) to pass through while selectively repelling other macromolecules [143]. Therefore, a rather high permselectivity of g-C_3_N_4_ membranes can be easily achieved. There is no doubt that g-C_3_N_4_ is a photocatalytic water treatment membrane with high separation efficiency and self-cleaning ability due to its unique structural features and excellent catalytic activity.

Wang et al. [144] fabricated a membrane with artificial nanopores and self-supporting spacers by assembling 2D g-C_3_N_4_ nanosheets into stacks with fine structures. The membrane exhibited considerable separation performance at a level of 29 L m^−2^ h^−1^ bar^−1^ with an 87% rejection of 3 nm molecules. The artificial nanopores in g-C_3_N_4_ nanosheets and the spaces created by unexfoliated segments between nanosheets provide ultra-low friction nanochannels for water transport. Huang et al. [145] revealed for the first time that g-C_3_N_4_ has a bactericidal effect on *E. coli* in water under visible light irradiation. Advanced photocatalytic membrane technology provides a new strategy to eliminate membrane fouling problems in current membrane technologies. Therefore, the introduction of g-C_3_N_4_ can endow water treatment membranes with catalytic degradation and antifouling properties which are more advantageous than conventional membranes. The hybridization of g-C_3_N_4_ nanosheets with other nanomaterials (such as GO, TiO_2_, Ag, AgBr, etc.) enhances self-cleaning ability due to the synergistic effects [146,147,148].

### 4.6. Others

Although two-dimensional material films have made significant progress, the related research reports on water treatment such as layered double hydroxides (LDHs) and hexagonal boron nitride (h-BN) are limited. Layered double hydroxide (LDHs) is an inorganic layered compound with a unique structure consisting of positively charged brucite-like two-dimensional sheets, charge-compensating anions, and solvated molecules located in interlayer channels. LDHs can be fabricated in a one-step process under hydrothermal conditions with compositional flexibility and tunability of interlayer channel distances resulting in functionally diverse smart membranes [149,150]. Hexagonal boron nitride (h-BN) nanosheets are isostructural with graphene, hence the name “white graphene”, and exhibit strong water treatment properties. However, due to the poor dispersibility of h-BN in water, which limits its utilization in membrane applications, it is usually functionalized [151,152,153].

## 5. Conclusions and Future Outlook

In summary, this review has highlighted the evident promise of two-dimensional nanomaterials (2DNMs) as emerging membrane materials for next-generation liquid separation technologies, especially in desalination, water purification and ion separation with extraordinary permeation, high selectivity, and excellent mechanical strength. A comparison of several state-of-the-art NF membranes and 2D material membranes recently reported in the literature is shown in Table 2, regarding their selectivity and water permeability as well as operating conditions. Significantly, 2DNM-based membranes with well-defined transport channels and ultrathin thickness have demonstrated extraordinary performance in molecular and ion separation. Despite promising results in experimental studies, there are still many challenges and unsolved mysteries for the scientific community and scholars. For atomic-thick 2D nanosheet membranes with artificial perforations or natural nanopores, experimental studies mainly focus on ion selectivity and permeation flux, as well as perforation methods and scale-up. For example, when single-layer graphene is produced by chemical vapor deposition, tearing and inherent defects will inevitably occur. At this point, multilayer nanoporous membranes can be produced to avoid defects, but the interactions between nanopores on different layers are still unclear. Membrane fouling is also common in the separation process, which affects the sustainable use of separation membranes. The effect of membrane fouling on ultrathin porous nanosheet membranes has not been investigated so far. Given that these aspects have yet to be thoroughly investigated and scientifically explained, the choice of layered membranes for developing large-scale applications appears to be a reliable strategy.

Although rapid growth in interest and a large number of achievements have been made in 2D laminar membranes, applying these ultrathin membranes in practical separation remains a technical challenge in this emerging field. Nanosheets can usually be produced using top-down exfoliation, and 2D laminar membranes can be prepared by vacuum filtration or coating methods. The performance of layered membranes may not be comparable to commercial nanofiltration membranes, thus requiring intercalation adjustment of the interlayer spacing by using different polymers or crosslinking agents. Moreover, the performance of state-of-the-art layered stacks is far from the theoretically predicted values, which may be due to the insufficient understanding of the structure–property relationships of the complicated nanochannels. The 2D layered membrane is easily swelled in water, which severely limits its wide applications. Additionally, current challenges that restrict the wide implementation of 2DNM-based membranes include the limited available techniques for exfoliating the high aspect ratio and defect-free nanoporous monolayers from bulk crystals, and drilling uniform, controllable, large-area, nanopores in graphene nanosheets, together with how to scale such ultrathin membranes into applicable separation devices.

Future studies may focus on exploring the emerging 2DNM-based membrane platforms by introducing new kinds of 2D materials that have already shown success in other related fields. The updated theoretical model and in-depth characterization techniques should be studied to accurately describe the particularly confined transport behavior through 2DNM-based membranes. Significant effort should be applied to develop robust membranes with stable performance under realistic operating conditions. More research is needed to address specific requirements for various exciting, yet challenging, applications, such as desalination and fuel cells.

## Figures and Tables

**Table 1:** Comparison of properties of 2D material membranes and their advantages and disadvantages in water treatment.

2DNM Membranes	Interlayer Spacing/Pore Size (nm)	Zeta Potential (mV)	Surface Area (m^2^/g)	Advantages	Disadvantages
GO	0.85	−24 to −46	90	Atomic thickness; Easy to synthesize; Oxygen-containing functional groups.	Membrane swelling; Unstable in aqueous environment.
rGO	0.7–1.2 nm	−24	130	Narrower nanochannels; Lower swelling; More stability.	Low permeability; Membrane swelling.
MXene (Ti_3_C_2_T_x_)	0.35 nm	−35	108	High permeability; Abundant surface groups; Natural hydrophilicity.	Low separation accuracy; Relatively single separation function; Membrane swelling.
MoS_2_	0.65 nm	−45	165	Rigid nanosheet; Long-term stability; Medium permeability.	Hydration of membrane is required at all times for efficient water transport; Requires functionalization.
MOF	<2 nm	-	-	High surface areas; Regular and highly tunable pore structure; Functional surface groups.	Instability; Difficulty for regeneration.
COF	1–3 nm	-	-	Inherent porosity; Ordered channel structure; Large surface area; Abundant hydrogen bonding sites.	Insoluble; Unprocessable microcrystalline powders.

**Table 2:** Comparison of separation performance of several two-dimensional material membranes and NF membranes.

Membrane	Permeability (L m^−2^ h^−1^ bar^−1^)	Rejection (%)	Feed Concentration (g L^−1^)	Operating Pressure (bar)	Ref.
NF-270 (polyamide)	11.6	Na_2_SO_4_: 94.0	2	10	[154]
NF-PDA/PEI/PAA	5.5	Na_2_SO_4_: 98.3	1	5	[155]
PIP-TMC/NTSC	10.6	Na_2_SO_4_: 97.8	0.5	5	[156]
MOF-303	0.74	Na_2_SO_4_: 96.0	1	5	[28]
UiO-66-NH_2_/TFN	30.8	Na_2_SO_4_: 97.5	1	4	[29]
TpTG_Cl_@CNFs	42.8	Na_2_SO_4_: 96.8	1	2	[43]
ACOF-1/HPAN	142	CR: 99.2	0.2	4	[42]
FNG/PES	131	MR > 90	0.01	1	[93]
GO/g-C_3_N_4_	33.5	Na_2_SO_4_: 92.5	2.8	1–5	[88]
ZIF-8@f-GOm	49.8	Na_2_SO_4_: 52.9	1	2	[90]
ZnO/rGO	225	MB: 98	0.025	1	[106]
rNPGO	63.06	Na_2_SO_4_: 92.9	2.8	6	[109]
Fe(OH)_3_/MXene	1084	EB: 90	-	1	[121]
GO/AA-Ti_3_C_2_T_x_	115.5	CR: 99.1	0.01	1	[122]
Ag@MXene	387.05	RhB: 79.93	0.05	1	[128]
MoS_2_/PVDF	24.7	MB: 82.17	0.37	1.38	[136]
g-C_3_N_4_/AAO	29.5	EB: 87.2	0.2	-	[144]
MWCNTs/BNNSs/GO	30.2	TCH: 96.1	0.03	0.9	[153]
